# Supplementary material for: MetaRibo-Seq measures translation in microbiomes
Source: Nat Commun. 2020 Jun 29;11:3268. doi: 10.1038/s41467-020-17081-z (PMC7324362; doi:10.1038/s41467-020-17081-z)
Supplement: Supplementary file 10 — Supplementary Data 7 [file 41467_2020_17081_MOESM10_ESM.zip › File2/Confidence_High_Taxonomy/58530_out.krona.html]

Javascript must be enabled to view this page.

members
magnitude
magnitudeUnassigned
count
unassigned
taxon
rank

58530\_out

63

63
superkingdom
2

976
phylum
63

class
63
200643

171549
63
order

171552
62
family

62
genus
838


SRS100907\_contig\_number\_7219
species
1
1947118

165179
42
species

SRS011134\_contig\_number\_43717SRS012849\_contig\_number\_1135SRS013638\_contig\_number\_7059SRS014415\_contig\_number\_1166SRS017307\_contig\_number\_contig-100\_558.176894SRS017375\_contig\_number\_353SRS018888\_contig\_number\_3971SRS019285\_contig\_number\_48SRS022609\_contig\_number\_42931SRS024132\_contig\_number\_24170SRS043667\_contig\_number\_20SRS043841\_contig\_number\_1298SRS044535\_contig\_number\_2460SRS045713\_contig\_number\_16116SRS046502\_contig\_number\_14326SRS047433\_contig\_number\_12588SRS050752\_contig\_number\_24801SRS053398\_contig\_number\_4247SRS054059\_contig\_number\_68SRS058145\_contig\_number\_contig-100\_640.35243SRS064757\_contig\_number\_14836SRS065397\_contig\_number\_4536SRS077194\_contig\_number\_20349SRS077641\_contig\_number\_77SRS077849\_contig\_number\_20452SRS077991\_contig\_number\_contig-100\_123.124SRS103987\_contig\_number\_183SRS103987\_contig\_number\_2377SRS104036\_contig\_number\_1458SRS1041031\_contig\_number\_contig-100\_240.142185SRS1041116\_contig\_number\_2903SRS1041134\_contig\_number\_273SRS1041137\_contig\_number\_13754SRS104327\_contig\_number\_3906SRS143181\_contig\_number\_193SRS144297\_contig\_number\_8151SRS144537\_contig\_number\_19868SRS144603\_contig\_number\_14416SRS144714\_contig\_number\_13183SRS146764\_contig\_number\_15150SRS148253\_contig\_number\_13893SRS150029\_contig\_number\_2615

2293125

SRS013521\_contig\_number\_3030
1
species

species
7

SRS016541\_contig\_number\_17536SRS023914\_contig\_number\_25329SRS049712\_contig\_number\_12945SRS049995\_contig\_number\_17094SRS053356\_contig\_number\_11730SRS105082\_contig\_number\_131SRS143876\_contig\_number\_11643
1263102


SRS012989\_contig\_number\_contig-100\_1262.55962.55962SRS013098\_contig\_number\_28541SRS015782\_contig\_number\_14272SRS015794\_contig\_number\_11165SRS023526\_contig\_number\_22679SRS047044\_contig\_number\_2098SRS074586\_contig\_number\_contig-100\_151.55125SRS078419\_contig\_number\_13577SRS097958\_contig\_number\_11768SRS144506\_contig\_number\_35098SRS147614\_contig\_number\_4088
11
species
59823

815
1
family

genus
1
816


SRS049896\_contig\_number\_26876
1
species
28116
